# A Comparative Study of Fasting Gastric Volume in Diabetic and Non-diabetic Patients Undergoing Elective Surgeries Using Ultrasonography: A Prospective Observational Study

**DOI:** 10.7759/cureus.33959

**Published:** 2023-01-19

**Authors:** Yashwanth Paidimuddala, Vishnuvardhan Voleti, Ravi Madhusudhan

**Affiliations:** 1 Anesthesiology and Critical Care, Sri Devaraj Urs Medical College, Kolar, IND; 2 Anesthesiology, Sri Devaraj Urs Medical College, Kolar, IND

**Keywords:** pulmonary aspiration, gastroparesis, gastric ultrasonography, fasting, diabetes mellitus

## Abstract

Background: Gastric ultrasound can be used to evaluate the residual gastric volume (GV) and contents before anesthetizing a patient. Autonomic gastropathy in patients with diabetes increases the risk of pulmonary aspiration of gastric contents. Therefore, the present study was conducted to assess the fasting GV among diabetics and healthy individuals using point-of-care (POC) ultrasonography.

Materials and methods: This prospective observational study was conducted in a tertiary care hospital between January 2021 and February 2022. A total of 122 patients included in the study were divided into two groups: group D (n = 61) patients had a history of diabetes mellitus and group C (n = 61) patients were non-diabetics (control). Gastric ultrasound was performed in supine and right lateral decubitus (RLD) positions. The following parameters were measured: duration of fasting, craniocaudal (CC) diameter, anteroposterior (AP) diameter, cross-sectional area (CSA), and GV using CC and AP diameters.

Results: The mean age of the participants was found to be 46.60 ± 13.77 years with 51.6% female patients and 48.4% male patients. Among the patients, there was a significantly higher mean level of CC diameter, AP diameter, and CSA in the supine position in diabetics compared to controls (p < 0.05). Similarly, there was a significantly higher mean level of CC diameter, AP diameter, and CSA in the RLD position in diabetics compared to healthy individuals (p < 0.05). The GV was significantly higher in diabetics (9.96 ± 14.520) compared to healthy individuals (-8.991 ± 20.95; p < 0.05).

Conclusion: Fasting GV assessed using POC ultrasonography in diabetic individuals was higher when compared to non-diabetics after similar periods of fasting.

## Introduction

In the perioperative period, a severe complication with a high fatality rate is the pulmonary aspiration of gastric contents. Diabetics are more likely to suffer from gastropathy due to autonomic dysfunction. Therefore, these patients are more prone to have gastroparesis, which results in delayed gastric emptying and makes them vulnerable to having a higher risk of aspiration than healthy individuals. A cohort study analysis of 538 patients stated that the normal fasting time did not enable proper stomach emptying, resulting in a change in anesthetic induction [1,2].

To date, standard fasting guidelines for people with diabetes are still up for debate. The 2011 fasting guidelines by the European Society of Anaesthesiology (ESA) allow diabetics to adhere to similar requirements as healthy individuals. Whereas the American Society of Anesthesiologists (ASA) in 2017 came up with the conclusion that patients with concomitant diseases have prolonged stomach emptying time; hence, it does not necessitate sticking to the standard eight hours nil per oral (NPO) period and that it has to be changed [3].

Point-of-care (POC) gastric ultrasound has gained popularity in recent times. It has been adapted in the field of anesthesiology gradually to make the clinical decision before anesthetizing patients where the fasting status is uncertain and in emergency patients where surgery is mandated. The ability to measure stomach contents at the bedside in real time using ultrasound, which is widely accessible, has been demonstrated to be effective. USG can be done during the pre-induction assessment to assess the patients' fasting gastric volume (GV) and to decide if it is more than the advised safe limit because these individuals are susceptible to encountering insufficiently empty stomachs despite an acceptable fasting period [4].

With this knowledge, the current research was conducted to analyze fasting GV among diabetics and non-diabetics undergoing elective surgeries using POC ultrasound. There is sparse literature available, which has stated a considerable difference in quantified fasting GV among diabetic and non-diabetic individuals where standardized fasting guidelines were followed in both groups.

## Materials and methods

This was a prospective observational study conducted on 122 patients undergoing elective surgeries at a tertiary care center between January 2021 and February 2022. Institutional Ethics Committee clearance was obtained before starting the study (SDUMC/KLR/IEC/613/2020-21). Written informed consent was taken from either the patients or next of kin for participation in the study. Patients of either gender aged 18-65 years and belonging to the American Society of Anesthesiologists (ASA) physical status 1 and 2 were included in the study. A minimum of six years of diabetic history was required to enroll diabetic patients in the study. Patients taking drugs that affect gastric motility, patients with renal failure, hypothyroidism, obesity (BMI > 30kg/m2), connective tissue disorders, history of gastrointestinal surgery, parturients, and patients with in situ nasogastric (NG) tube were excluded from the study.

A total sample size of 122 patients was calculated based on a study by Garg et al., with 90% power, and an error of 0.05 with 61 patients in each group [5]. Patients scheduled for elective surgeries and satisfying the inclusion criteria were distributed into either group D having a history of diabetes mellitus or group C, which are non-diabetics (control). Diabetic patients were evaluated for the duration of type 2 diabetes mellitus, drug history, control of blood glucose, and symptoms of gastropathy. A proper physical examination followed by a systemic examination was done and relevant investigations were checked. NPO status was evaluated and fasting duration was noted.

Preoperative bedside gastric USG was performed by an anesthesiologist who was unaware of the study population groups, using Philips InnoSight (Philips Ultrasound Inc., Reedsville, PA) low frequency, curvilinear transducer probe (2-6 MHz). Initially, study participants were positioned in the supine position and then switched to the right lateral decubitus (RLD) position later. Anteroposterior (AP) and craniocaudal (CC) diameters were measured in the RLD position and cross-sectional area (CSA) was estimated using the following formula: CSA = (AP × CC × π)/4. GV was estimated using Perlas equation as follows: GV (ml) = 27.0 + 14.6 × right‑lat CSA - 1.28 × age [6].

## Results

**Figure 1:**
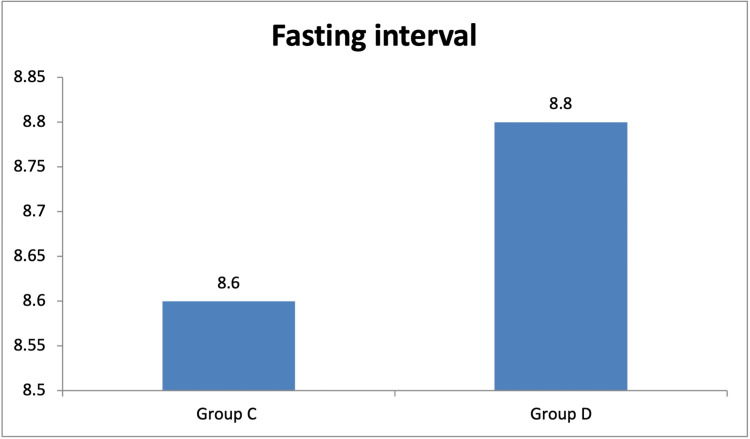
Comparison of the mean duration of fasting between the groups Group C = controls; Group D = diabetics. Fasting interval in hours.

The mean age of the participants was found to be 46.60 + 13.77 years. Among the participants, 51.6% were females and 48.4% were males, with marginal female preponderance. The mean age in years in group C was 45.5 + 15.5 and it was 51.7 + 11.0 in group D. The gender distribution was as follows: in group C, 31 (50.8%) participants were females and 30 (49.2%) were males, whereas in group D, 32 (52.5%) participants were females and 29 (47.5%) were males. The duration of the fasting interval (Figure 1) in group C was 8.6 + 0.9 hours and it was 8.8 + 0.9 hours in group D, which is almost similar.

**Figure 2:**
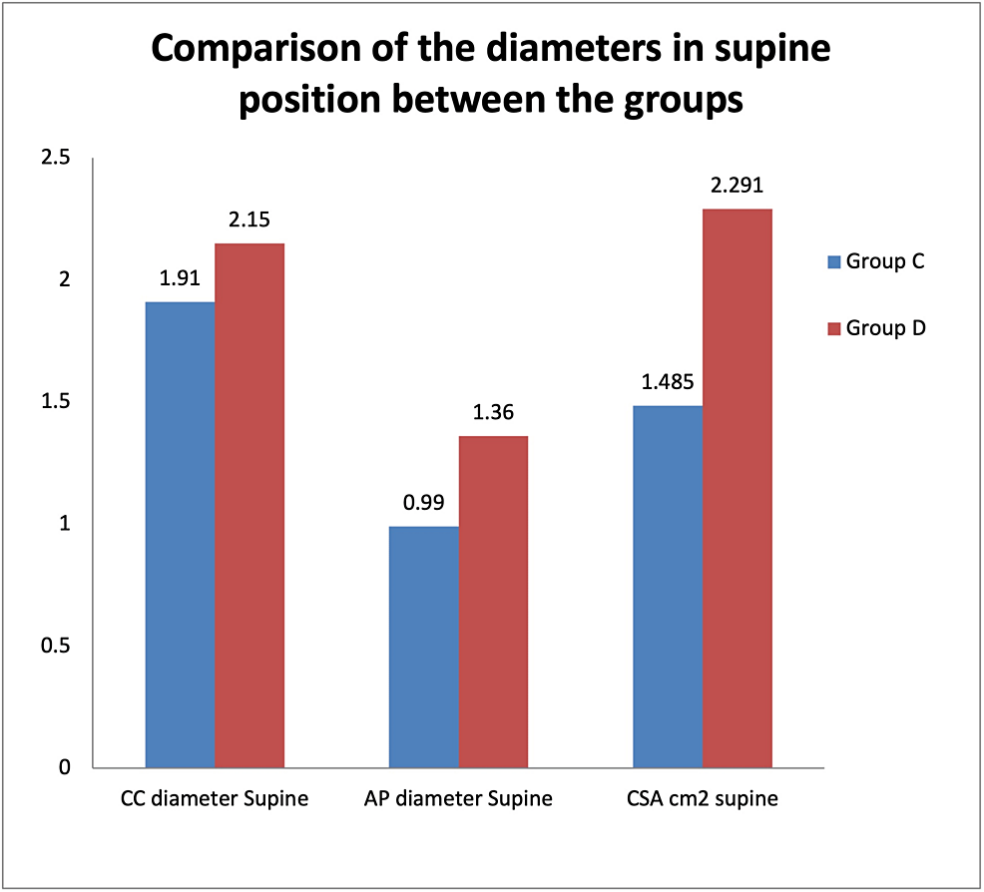
Comparison of the diameters in the supine position between the groups CC = craniocaudal; AP = anteroposterior; CSA = cross-sectional area in cm^2^; Group C = controls; Group D = diabetics.

The diameters measured in the supine position (Figure 2) were as follows: group C illustrates the CC diameter of 1.91 + 0.19, AP diameter of 0.99 + 0.10, and CSA of 1.485 + 0.212, and in group D, the CC diameter was 2.15 + 0.11, AP diameter was 1.36 + 0.07, and CSA was 2.291 + 0.180. Among the groups, there was a significantly higher mean level of CC diameter, AP diameter, and CSA in group D when compared to group C.

**Figure 3:**
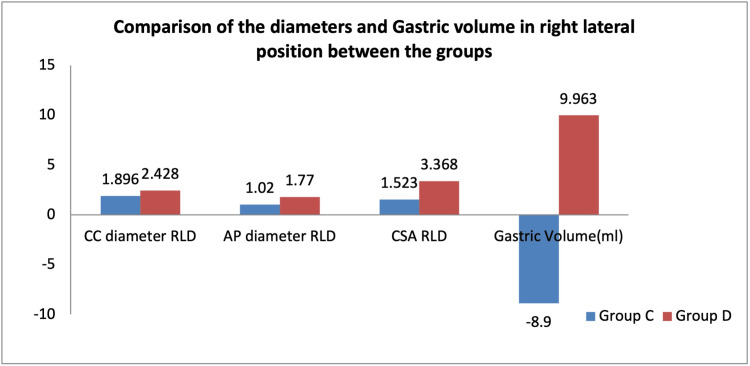
Comparison of the diameters and gastric volume in the right lateral position between the groups CC = craniocaudal diameter in centimeters; AP = anteroposterior diameter in centimeters; CSA = cross-sectional area in cm2; GV = gastric volume in milliliters; Group C = controls; Group D = diabetics; RLD: right lateral decubitus.

The diameters measured in the RLD (Figure 3) position were as follows: group C illustrates the CC diameter of 1.89 + 0.072, AP diameter of 1.02 + 0.14, and CSA of 1.523 + 0.202, and in group D, the CC diameter was 2.42 + 0.12, AP diameter was 1.77 + 0.12, and CSA was 3.36 + 0.286. Among the groups, there was a significantly higher mean level of CC diameter, AP diameter, and CSA in group D when compared to group C (p < 0.05). The calculated mean GV in RLD was -8.9 + 20.554 in group C whereas it was 9.963 + 14.520 in group D.

## Discussion

Diabetes mellitus is contemplated as a high-risk condition that constitutes a deliberate challenge to anesthetists in various aspects. The most dreaded complication is the aspiration of gastric contents since diabetics are likely to be full stomachs because of autonomic gastropathy [7]. In present practice, NPO status is obtained through patients, which is inaccurate, and in those at higher risk for delayed stomach emptying, it may provide a larger risk for aspiration. Prior to anesthetic induction, utilization of POC gastric ultrasonography as a screening tool can help in decision-making and minimize unnecessary perioperative complications [8]. USG is readily accessible and is shown to be a viable bedside evaluation method to assess stomach contents in real time. USG was performed before induction to assess patients' fasting GV to see whether it is greater than the suggested safe limit [4]. The mean age of the participants was found to be 46.60 ± 13.77 years. Among the participants, 51.6% were female patients and 48.4% were male patients, with marginal female preponderance in the present study. The mean age of patients and gender distribution between the groups was found to be comparable. Similar to the present study, Cunha et al., documented the similar mean age between the groups. The diabetes group had a mean age of 49.3 ± 16.4 years, whereas the nondiabetic group had a mean age of 49.4 ± 16.8 years [8].

In a study by Sharma et al., the majority of the patients were in the age of 60 years with 57% male and 43% female patients [9]. In Cunha et al.'s study, the mean age in the diabetic group was 49.3 ± 16.4 years, and in the non-diabetic group, it was 49.4 ± 16.8 years, with 68% male and 32% female patients [8]. Acute intraoperative aspiration of stomach contents is a feared and possibly deadly complication linked with morbidity from pneumonitis or aspiration pneumonia. The amount, type, and acidity of the gastrointestinal contents can all affect the degree of lung damage. Sonography in both the lying down and RLD position among patients is critical for adequate risk classification. In the supine position, the antrum may look empty, but it may seem full in the RLD position. The visible rise in GV in the RLD position is most likely because stomach contents tend to the gastric outlet [2,10].

Fasting duration was comparable between both groups. There was a significantly higher mean level of CC diameter, AP diameter, and CSA in the supine position in diabetic patients compared to healthy controls (p < 0.05). Similarly, there is a significantly higher mean level of CC diameter, AP diameter, and CSA in RLD position in diabetic patients compared to healthy individuals (p < 0.05). The GV was significantly higher in cases (76.16 ± 4.18) compared to the controls (49.23 ± 2.95) in the study (p < 0.05).

Similar to the present study, Rajeswari documented that in both the right lateral and semi-recumbent positions, diabetic individuals exhibited significantly higher mean antral CSA and estimated mean gastric residual volume (GRV). In both the right lateral (p = 0.0001) and semi-sitting postures, the gastric antrum looked empty in a considerably larger frequency of non-diabetic individuals than in diabetic patients [11]. Another study by Haramgatti et al. documented that despite the disparity in CSA and GV among diabetic and non-diabetic groups, both revealed a minimal residual GV (<1.5 ml/kg). The NG tube aspirate in non-diabetic and diabetics was 0.3 ± 0.78 ml and 1.24 ± 1.46 ml, respectively, and the difference was significant [12]. When compared to non-diabetic individuals, those with chronic diabetes had larger residual GV and antral CSA. The clinical importance of these results requires further data before specific advice for diabetes individuals can be developed.

A study by Sharma et al. documented that diabetics and chronic kidney disease patients demonstrated a statistically significant increase in CSA among supine and RLD positions. They discovered a rise in calculated stomach capacity when patients' BMI rose [9]. Patients with chronic diabetes resulted in higher residual GV in comparison to healthy individuals after eight NPO hours for elective surgeries in a study conducted on 25 diabetes mellitus patients with six years of history and 25 normal healthy controls on ultrasound. Sabry et al. found that compared to the control group, the diabetic group had a greater median antral CSA and a higher estimated stomach residual volume. In addition, the diabetic group had a larger aspirated GV through the nasogastric tube than the control group [13]. The association between computed residual stomach volume using ultrasonic measurements and the volume of aspirated gastric contents via NG tube was extremely excellent. After fasting for eight NPO hours for an elective surgical procedure, chronic diabetics had a greater residual GV than healthy controls.

Diabetics had a larger mean volume of stomach aspirate when compared to non-diabetics. In individuals with long-standing diabetes, the current fasting guidelines for elective surgery are inconclusive. As a result, we recommend that POC ultrasound can be used as an effective screening tool to assess aspiration risk and tailor anesthetic management. While current fasting recommendations are acceptable for healthy people, they are insufficient in patients with risk factors. In individuals with risk factors, ultrasound measurement of preoperative stomach capacity is a useful screening technique. Sharma et al. discovered that fasting for 10 hours did not ensure an empty stomach and that comorbidities such as diabetes made patients more likely to have hazardous gastric contents when utilizing bedside gastric USG on adult patients coming for elective surgery [9]. Following the current preoperative fasting guidelines, about 50% of type 2 diabetes patients have a full stomach. The use of preoperative ultrasonography to measure stomach content in type 2 diabetes patients is recommended [14].

Our study found out that NPO > 6-10 hours will not ensure a bare stomach, independent of comorbid conditions. As a result, it is obvious that bedside ultrasonography may be utilized to assess the status of stomach contents and may be used to stratify aspiration risk. It could be useful in a variety of therapeutic situations where the risk of aspiration is unknown or uncertain.

Our study had a few limitations. Firstly, the sample size was relatively small to draw conclusions. Also, the effect of obesity on fasting GV was not evaluated, as obesity coexists in diabetics and can be a confounding factor. The relation between the duration of diabetes mellitus and fasting GV was not studied. We did not study the incidence of pulmonary aspiration of gastric contents in our study participants in whom there was enhanced residual GV.

## Conclusions

Antral CSA and thereby fasting GVs, measured using POC ultrasonography in the RLD position, were significantly higher in patients suffering from type 2 diabetes mellitus compared to healthy individuals of similar age groups. This quantitative assessment of gastric contents using POC ultrasound is a valuable tool in identifying patients at risk of pulmonary aspiration of gastric contents and helps the perioperative physicians in decision-making. Further studies with larger sample sizes and having patients with associated comorbidities, such as obesity, should be conducted to evaluate the effect of factors that delay gastric emptying in diabetic patients, who are subjected to elective surgeries under anesthesia.
